# Novel insights which may translate into treatments for irritable bowel syndrome

**DOI:** 10.3389/fphar.2013.00160

**Published:** 2013-12-31

**Authors:** Angelo A. Izzo

**Affiliations:** Department of Pharmacy, University of Naples Federico IINaples, Italy

**Keywords:** 5-hydroxytryptamine, brain-gut axis, cannabinoid receptors, enkephalinase inhibitors, irritable bowel syndrome, kynurenine pathway, pharmacotherapy, toll-like receptors

Irritable bowel syndrome (IBS) is a very common functional disorder of the digestive tract. Despite the prevalence and the social impact of IBS, the exact etiopathogenesis is incomplete and the pharmacological treatment is unsatisfactory. In this research topic, *Frontiers in Pharmacology* brings together a group of review and original articles focusing on IBS.

Fabrizio De Ponti brilliantly outlined the drugs currently used in—or in development for—IBS, within a scenario in which the specific armamentarium of medications is unsatisfactory (De Ponti, 2013). Jakub Fichna and Martin Storr focused their review article on the disturbances in the brain-gut axis as possible cause of IBS (Fichna and Storr, 2012). The Authors illustrated the pathophysiological mechanisms which contribute to the generation of IBS symptoms, with a special emphasis to stress, emotion and psychological factors. The future of anti-IBS drugs targeting the brain-gut axis were also highlighted. The bidirectional communication between the brain and the gut opens up new treatment possibility for IBS patients with mood disturbances that are refractory to first-and second-lines therapies.

Jeremy Gale and Lesley Houghton reviewed the preclinical and clinical data which support the potential use of gabapentin and pregabalin in disorders characterized by visceral hypersensitivity, such as IBS (Gale and Houghton, 2011). Both gabapentin and pregabalin bind with high affinity to alpha 2 delta subunits of voltage-gated calcium channels and inhibit both visceral nociception and motility. Although limited, clinical studies on visceral pain are in agreement with animal results and support a continued research and development of the alpha 2 delta ligands in IBS.

Eberlin et al. reviewed the pharmacokinetics, pharmacodynamics and clinical data of racecadotril, a powerful and selective enkephalinase inhibitor, which has emerged as a promising drug in the antisecretory therapy (Eberlin et al., 2012). In multiple direct comparative studies, racecadotril was at least as effective as loperamide in the treatment of acute diarrhoea, and exhibited significantly better tolerability. Although the results are robust and encouraging, the potential of racecadotril in D-IBS patients remains to be established.

A pan-Irish study investigated the consequences of toll-like receptors (TRLs) activation on the production of kynurenine (i.e., one of the metabolites of the kynurenine pathways derived from tryptophan) in IBS (Clarke et al., 2012). Whole blood from IBS patients and healthy controls was cultured with a number of TLR agonists. Tryptophan and kynurenine were assayed, by HPLC, both in the plasma and in cell culture supernatants. IBS subjects had an elevated plasma kynurenine:tryptophan ratio compared to healthy controls, which is suggestive of tryptophan metabolism via the kynurenine pathway. Furthermore, a differential downstream profile of kynurenine production subsequent to TLR activation in IBS patients - compared to healthy controls – was demonstrated. Collectively, such results suggest (1) that a pharmacological modulation of TLRs, by controlling the abnormal kynurenine pathway, be a novel potential strategy for IBS and (2) the use of plasma tryptophan metabolites assay as a biomarker for IBS diagnosis.

Beattie et al. investigated the *in vitro* and *in vivo* pharmacodynamic properties of a novel 5-HT_4_ receptor agonist, namely TD-8954 (Beattie et al., 2011). TD-8954 was found to be a potent (*pKi* = 9.4) and selective (>2000-fold over the all other 5-HT receptors and over a plethora of receptors, enzymes, and ion channels) 5-HT_4_ receptor agonist *in vitro* with strong *in vivo* gastrointestinal activity in guinea pigs, rats, dogs, and in healthy humans. TD-8954 may have value in the treatment in C-IBS sufferers.

Interleukin-6 (IL-6) is elevated in the plasma of D-IBS patients (Rana et al., 2012) and IL-6 gene polymorphisms may change individual susceptibility to IBS (Barkhordari et al., 2010). O'Malley et al. evaluated the effect of IL-6 on colonic ion transport (a measure of intestinal absorption and secretion) in the distal colon of the Wistar Kyoto rat (WKY) (O'Malley et al., 2012), a pre-clinical model for IBS. In colonic sheets mounted in Using chambers, IL-6 evoked secretion preferentially in WKY colons (as compared to control rats) and this effect was believed to be due to increased sensitivity of submucosal neurons to the pro-inflammatory cytokine. Such results bolsters our knowledge of IL-6 as a contributory factor in the pathophysiology of IBS.

Cannabinoids—via CB_1_ and/or CB_2_ receptor activation—exert pharmacological actions which are potentially beneficial in IBS (Izzo and Coutts, 2005; Storr et al., 2008). Kimball et al. evaluated the effect of selective cannabinoid receptor agonists in a mouse model of accelerated upper gastrointestinal transit resembling post-inflammatory IBS (PI-IBS) (Kimball et al., 2010). The experimental model is generated by intracolonic administration of mustard oil, which induce transient colitis and, in the longer term (i.e., 28 days after mustard oil) a functional increase in gastrointestinal transit that is observed when there is no inflammation (Kimball et al., 2005). It was found that both cannabinoid receptor subtypes were up-regulated in the small intestine, an effect closely associated to increased efficacy of both CB_1_ and CB_2_ receptor agonists in normalizing the accelerated transit (Kimball et al., 2010). These results suggest that the altered cannabinoid CB_1_ and CB_2_ responsiveness is maintained long after an initial inflammatory period and suggest a role of cannabinoid receptors in the underlying pathophysiology of PI-IBS.

The differentiated Caco-2 cells intestinal cell line, derived from a human colon carcinoma has been used for drug absorption studies as well as to evaluate the epithelial barrier integrity in relation to IBS as well as to investigate the intestinal permeability of IBS drugs (Catalioto et al., 2008; Piche et al., 2009). Within our research topic on IBS, Kauffman et al. compared two alternative sources of human intestinal cells, i.e., commercially available primary human intestinal epithelial cells (hInEpCs) and induced pluripotent stem cell (iPSC)-derived intestinal cells, to Caco-2, for possible use in intestinal transport assays (Kauffman et al., 2013). Measurements of intestinal marker expression, formation of monolayers with tight junction formation and functional molecule transport and binding revealed that primary hInEpCs and iPSC-derived intestinal cells could offer an alternative source of human intestinal cells for understanding intestinal epithelial patho(physiology) and drug transport.

In summary, this research topic should provide a useful resource for IBS researchers, both basic and clinical. The pharmacological studies, together with new strategies for drug discovery, highlight that more research is urgently required to translate novel and innovative basic concepts into prescribing options for healthcare professionals. It is hoped that this collection of articles will inspire further research into IBS.

## Abbreviations

5-HT: 5-hydroxytryptamine
C-IBS: constipation predominant irritable bowel syndrome
D-IBS: diarrhoea predominant irritable bowel syndrome
hInEpCs: human intestinal epithelial cells
IBS: irritable bowel syndrome
IL-6: interleukin-6
iPSC: induced pluripotent stem cell
PI-IBS: post-inflammatory irritable bowel syndrome
TLRs: toll-like receptors
WKY: Wistar Kyoto rat.
